# Bactericidal Effect and Mechanism of Polyhexamethylene Biguanide (PHMB) on Pathogenic Bacteria in Marine Aquaculture

**DOI:** 10.3390/biology14050470

**Published:** 2025-04-25

**Authors:** Lanting Wu, Chunyuan Wang, Yingeng Wang, Yongxiang Yu, Zheng Zhang, Cuiping Ma, Xiaojun Rong, Ling Chen, Meijie Liao, Yapeng Yang

**Affiliations:** 1 College of Biological Engineering, Qingdao University of Science and Technology, Qingdao 266042, China; w13988337725@163.com (L.W.); mcp169@163.com (C.M.); 2 State Key Laboratory of Mariculture Biobreeding and Sustainable Goods, Yellow Sea Fisheries Research Institute, Chinese Academic of Fishery Sciences, Qingdao 266071, China; wangcy@ysfri.ac.cn (C.W.); yuyx@ysfri.ac.cn (Y.Y.); zhangzheng@ysfri.ac.cn (Z.Z.); rongxj@ysfri.ac.cn (X.R.); liaomj@ysfri.ac.cn (M.L.); 13523963716@163.com (Y.Y.); 3 Laboratory for Marine Fisheries Science and Food Production Processes, Laoshan Laboratory, Qingdao 266237, China; 4 Shandong Center for Quality Control of Feed and Veterinary Drugs, Jinan 250109, China; 13708935340@163.com

**Keywords:** mariculture, polyhexamethylene biguanide (PHMB), bactericidal effect, bactericidal mechanism, *Vibrio*, *Bacillus*

## Abstract

Guanidine disinfectants are cationic polymers recognized for their effective sterilization properties and their ability to prevent bacterial resistance. As a result, they are widely utilized in medical, healthcare, household, and animal husbandry settings. However, the bactericidal effects and mechanisms of guanidine in marine aquaculture systems remain unclear due to the polymeric nature of guanidine ions and the complexity of marine environments. The inhibitory effects and bactericidal mechanisms of polyhexamethylene biguanide (PHMB) on key pathogens and probiotics are examined in this study. It was shown that PHMB had inhibitory effects on *Vibrio parahaemolyticus* (VP), *Photobacterium damselae* subsp. *damselae* (PDD), *Bacillus subtilis* (BS), *Escherichia coli* (EPEC), and *Staphylococcus aureus* (SAU), with minimum inhibitory concentrations (MICs) ranging from 3.91 to 125.0 µg/mL, and minimum bactericidal concentrations (MBCs) from 15.63 to 250.0 µg/mL. A stronger bactericidal effect of PHMB on marine bacteria compared to EPEC and SAU was exhibited. It was shown in ion interference experiments that the addition of calcium ions reduced the bactericidal effectiveness of PHMB against VP and PDD by 87.73% and 53.35%, respectively. At a PHMB concentration of 62.50 µg/mL, minor changes in cell surface potential energy (CSPE) were exhibited by Gram-positive bacteria (SAU and BS), while more significant alterations were shown by Gram-negative pathogens. It was revealed by propidium iodide staining and scanning electron microscopy (SEM) analysis that the bacterial cell membrane was directly disrupted by PHMB. DNA and RNA release analysis further revealed that following PHMB treatment, changes in membrane permeability were exhibited by Gram-negative pathogens, with a significant increase in extracellular DNA content as PHMB concentration increased. No such effect was observed in Gram-positive bacteria. Additional evidence was provided by the findings that PHMB effectively inhibits bacterial pathogens in mariculture systems, with a significantly stronger inhibitory effect on Gram-negative pathogens than on Gram-positive bacteria. These results indicated that PHMB could serve as a new antimicrobial agent in mariculture.

## Simple Summary

The intensification of aquaculture practices elevates risks of pathogen transmission, necessitating advanced disinfectants. Polyhexamethylene biguanide (PHMB), a promising disinfectant, exhibits unclear seawater applicability and species-specific mechanisms against marine bacteria. This study demonstrated that PHMB exerts bactericidal effects by disrupting cell membrane permeability, yet its efficacy varies with bacterial characteristics. Notably, the ionic composition of seawater was identified as a potential limiting factor, particularly through calcium-mediated interference in PHMB’s activity against marine pathogens (such as *Vibrio parahaemolyticus* and *Photobacterium damselae* subsp. *damselae*). These findings provide critical insights into optimizing PHMB-based disinfection strategies in aquaculture systems.

## 1. Introduction

The aquaculture industry is currently one of the fastest-growing global sectors, accounting for 47% of the world’s aquatic animal husbandry [1]. This increased demand for production and consumption has led to large-scale and intensive farming facilities [2], necessitating overcrowded farming practices that has accelerated the proliferation of pathogenic microorganisms, resulting in a higher incidence of diseases and increased mortality of farmed animals [3]. The most common treatments for these problems are via the use of disinfectants, antibiotics, herbal substitutes, as well as vaccines. In particular, highly effective guanidine disinfectants and cationic surfactants are widely used in the pharmaceutical and food industries, as well as in disinfecting plastics, fabric softeners, paints, swimming pools, and paper [4].

Seawater contains a diverse range of bacteria, including both pathogenic and beneficial species. This has sparked significant interest in the effectiveness of disinfectants in eliminating various bacteria in aquaculture environments. *Vibrio parahaemolyticus* (VP) is a Gram-negative halophilic bacterium that is widespread in marine and brackish water environments and can infect a variety of marine animals [5]. Certain strains of this bacterium are pathogenic not only to fish and crustaceans (e.g., shrimp and crabs) but also to humans [6,7]. *Photobacterium damselae* subsp. *damselae* (PDD) is a Gram-negative bacterium that infects fish, crustaceans, mollusks, and cetaceans [8]. Gram-positive *Bacillus* bacteria possess antibacterial and anti-biofilm properties, display rapid growth, and possess minimal nutritional requirements [9]. These properties make them suitable as probiotics provided they meet the FAO/WHO criteria [10]. Numerous *Bacillus* strains have been isolated from marine environments that have demonstrated anti-vibrio activity [11]. Accumulated scientific evidence has confirmed the purification effect of *Bacillus* on pond water and this has led to its widespread use in aquaculture [12].

Polyhexamethylene biguanide (PHMB) is a cationic polymer with potent antibacterial activity against both Gram-positive and Gram-negative bacteria. The polymer binds to the negatively charged bacterial cell membranes, causing cytoplasmic leakage and bacterial death [13]. Guanidine disinfectants have been utilized in the aquaculture industry and PHMB was found to be effective against *Vibrio parahaemolyticus*, which is the causative agent of acute hepatopancreatic necrosis disease (AHPND) in shrimp [14]. PHMB exhibited its strongest inhibitory activity at the cell membrane due to the net negative charge of the bacterial outer membrane. This negative charge originates from phosphoric or lipoteichoic acids in the cell wall of Gram-positive bacteria or from lipopolysaccharides (LPS) and phospholipids in the outer membrane of Gram-negative bacteria. The plasma membrane consists of phospholipids and embedded proteins that collectively confer a negative charge to the membrane [15,16]. Due to the positive charge of PHMB, its cationic amino group interacts with the anionic phospholipids in the bacterial cell wall to exert an antibacterial effect [17,18]. Cationic fungicides exhibit varying bactericidal effects on different bacteria, likely due to differences in the surface charge of the cells [4,16].

This study first examined the bactericidal effects of PHMB on various aquatic pathogenic and beneficial bacteria. Secondly, we explored how different ions in the seawater environment influence PHMB’s bactericidal effect, aiming to provide a database for its application in seawater aquaculture. Finally, we compared the damage to bacterial cell membranes and changes in surface potential after PHMB treatment, assessing its differential effects on Gram-negative and Gram-positive bacteria. Additionally, we measured DNA and RNA leakage and observed leakage time after treatment at various PHMB concentrations to determine which bacteria exhibit faster cell membrane permeability changes and enhanced bactericidal effects. These findings will inform the application of PHMB in diverse aquaculture environments and the treatment of bacterial diseases, further elucidating its mechanism of action.

## 2. Material and Methods

### 2.1. Materials

Polyhexamethylene biguanide (PHMB) was obtained from Aladdin Chemical Reagents (P192452-100g) with a purity of ≥99% (Shanghai, China).

### 2.2. Experimental Bacteria

*Staphylococcus aureus* (SAU) and *Escherichia coli* (EPEC) were purchased from the China Industrial Microbiological Culture Collection Center (Beijing, China). Aquatic pathogenic bacteria strains of *Photobacterium damselae* subsp. *damselae* (PDD), *Vibrio parahaemolyticus* (VP), and aquatic *Bacillus subtilis* (BS) were obtained from the pathogen bank of mariculture in the Yellow Sea Fisheries Research Institute, Chinese Academy of Fisheries Sciences (Qingdao, China). The pathogenicity of these strains to aquatic animals has been verified [19].

### 2.3. Bacterial Solution Preparation

Cryopreserved bacterial strains were activated on TSB solid medium and all bacteria were always cultured under optimal growth conditions. Individual activated colonies of PDD, VP, and BS were picked and cultured in TSB liquid medium at 28 °C and 180 rpm until the bacterial concentrations were adjusted to 1.0 × 10^6^ CFU/mL and 1.0 × 10^8^ CFU/mL. Individual colonies of SAU and EPEC were selected and cultured in LB medium at 37 °C and 180 rpm. The bacterial concentrations were spectrophotometrically adjusted to 1.0 × 10^6^ and 1.0 × 10^8^ CFU/mL, respectively.

### 2.4. Determination of MIC and MBC

Minimal inhibitory concentration (MIC) and minimal bactericidal concentration (MBC) were determined by inoculation using 100 µL experimental strain (1.0 × 10^6^ CFU/mL) and 100 µL of medium (PDD, VP, and BS use TSB medium; EPEC and SAU use LB medium) to a 96-well plate containing serial dilutions of the test drug. The final drug concentrations were 250, 125, 62.5, 31.25, 16, 8, 4, and 2 µg/mL. In addition, 100 µL medium with 100 µL bacterial solution served as the negative control, 100 µL medium with 100 µL PHMB served as the positive control, while 200 µL of medium was used as the blank control. Each concentration was repeated in four replicates and 30 µL of 2, 3, 5-Triphenyltetrazolium chloride (1% solution; TTC) was added to each well as a chromogenic agent. When the negative control showed a color change, the lowest colorless concentration in the different concentration treatment groups of PHMB was recorded as MIC.

A sample of 100 µL from a drug treatment group with a concentration higher than the MIC was inoculated in a TSB plate. PDD, VP, and BS samples were incubated overnight at 28 °C and SAU and EPEC were incubated overnight at 37 °C. The minimum concentration at which bacteria do not grow in plates was MBC.

### 2.5. The Effect of Metal Ion Concentrations on the Bactericidal Activity of PHMB

Four metal ions (Na^+^, Ca^2+^, K^+^, and Mg^2+^) were selected to assess the impact of seawater ions on the bactericidal activity of PHMB. Based on the salt ion percentages in seawater, NaCl concentrations were set to 0, 5, 10, 15, 20, and 25 mg/mL, while the concentrations of CaCl_2_, KCl, and MgCl_2_ were set to 0, 1, 2, 3, 4, and 5 mg/mL. The ion and PHMB solutions were added to a 24-well plate to achieve the desired ion concentration gradient with the final PHMB concentration in all wells set to the MBC of each bacterium. The control group received no drug solution. Finally, 1.0 × 10^6^ CFU/mL of bacterial solution was added. Bacterial counts were performed by aspirating 100 µL and spreading it onto solid medium plates as per above. Bacterial counts were performed under different conditions and the bactericidal effectiveness of PHMB was then calculated. The experiment was performed with three replicates and the results were expressed as the mean ± standard deviation [20].

### 2.6. Zeta Potential Measurement

PDD, VP, as well as BS showed significant differential changes in zeta potential after treatment with PHMB above 62.5 µg/mL, while SAU and BS were not significantly different from the control in the concentration range of 250–15.63 µg/mL. Thus, 62.5 µg/mL was chosen as the experimental concentration.

Zeta potential analysis was determined by a Zetasizer Nano Zse device (Malvern, Worcestershire, UK). Readings were taken using 1 mL of the sample deposited in a capillary cell (DTS1070, Malvern, Worcestershire, UK).

The zeta potential of experimental strains treated with 62.5 µg/mL PHMB for 1 h was measured using laser Doppler velocimetry (Bacterial concentration was about 1.0 × 10^8^ CFU/mL). After PHMB treatment, the strains were washed and resuspended in saline for easier detection, and the non-PHMB-treated strains were used as controls.

### 2.7. Propidium Iodide (PI) Staining Experiments

PI staining was carried out as previously described [21]. In brief, the bacterial suspension (1.0 × 10^6^ CFU/mL) was pelleted by centrifugation at 5000 rpm for 5 min and pellets were then washed with PBS 2–3 times. PHMB solution was added to aliquots at 4×, 2×, 1×, 1/2× and 1/4 × MIC. After two hours of drug treatment, the cells were washed with PBS, resuspended and incubated in the dark with a 5% PI solution for 30 min for staining. Bacterial cells without PHMB treatment served as controls. After staining, 30 µL of the solution was added to slides and observed using fluorescence microscopy at an excitation wavelength of 510–560 nm. Three replications of the experiment were performed.

### 2.8. SEM Observation

The morphology of EPEC, SAU, VP, PDD, and BS with and without PHMB treatment was observed using scanning electron microscopy (SEM). The PHMB-treated group was exposed under 1 × MIC for 2 h. Observations were performed following the previously described methods [22]. The bacterial suspension (1.0 × 10^6^ CFU/mL) was treated with the antibacterial agents for 2 h; cells were collected and washed three times with PBS and fixed overnight at 4 °C using 2.5% glutaraldehyde. Following PBS washing, the cells were dehydrated with ethanol and then replaced twice with isoamyl acetate. Finally, the sample was fixed on the SEM carrier, sputtered with gold under vacuum, and observed using a Vega3 SEM (Tescan, Brno, Czech Republic).

### 2.9. Effect of PHMB on DNA and RNA Leakage

The effects of PHMB on cell permeability were examined as previously described [23]. The bacterial suspension was adjusted to 1 × 10^6^ CFU/mL before being washed and resuspended three times with PBS. Subsequently, the cells were treated with PHMB at concentrations of 2 × MIC, 1 × MIC, 1/2 × MIC, and 1/4 × MIC for 0 h, 2 h, 4 h, 6 h, and 8 h. Untreated bacterial cells served as the control. The culture medium was centrifuged at 4 °C and 5000 rpm for 10 min to obtain the supernatant. The release of DNA and RNA from the cytoplasm was measured at 260 nm using a Nano Drop 2000 UV–Vis spectrophotometer (Thermo Fisher Scientific Inc., Waltham, MA, USA).

### 2.10. The Prevention Effect of PHMB on Litopenaeus vannamei

Shrimp infected with VP acquire the infection orally. VP colonizes the hepatopancreas and intestines, leading to morbidity. In contrast, PDD infects fish through a similar oral route. In this study, we selected shrimp as the model organism and VP as the pathogenic agent to investigate the preventive effects of PHMB in shrimp aquaculture.

*L. vannamei* with a body length of about 10 ± 1 cm were cultured in well-oxygenated seawater at under 27 ± 0.5 °C. Each aquarium tank with 200 L of water, and 25 shrimp were cultured in each seawater tank. Fasting was prohibited before the experiment. *L. vannamei* were divided into five groups: the blank group, positive group, 4 µg/mL group, 2 µg/mL group, and 1 µg/mL group, with three replicates for each group. All groups, except for the blank control group, were subjected to bath treatment with VP at a concentration of 1 × 10^5^ CFU/mL. The positive control group received VP bath treatment alone, without the addition of PHMB. PHMB treatment groups were subjected to addition of PHMB at concentrations of 4 µg/mL, 2 µg/mL, and 1 µg/mL, respectively, at three parallels per group. The number of dead and surviving shrimp was counted daily for a total of seven days.

## 3. Results

### 3.1. Determination of MIC and MBC for Different Bacteria

We initially examined the effects of PHMB against five strains of representative bacteria and treatments exhibited varying effects at concentrations to achieve the MIC and MBC for each bacterium. The MICs of aquatic pathogens and beneficial bacteria ranged from 3.91 to 7.81 µg/mL while the MBCs ranged from 15.63 to 62.50 µg/mL. The MICs of EPEC and SAU were 31.25 and 125.00 µg/mL, respectively, with corresponding MBCs of 31.25 and 250 µg/mL, both of which were higher than those of the aquatic pathogens. PHMB exhibited a stronger bactericidal effect on marine bacteria compared to EPEC and SAU (Table 1).

### 3.2. Effect of Ions on the Bactericidal Activity of PHMB

**Figure 1:**
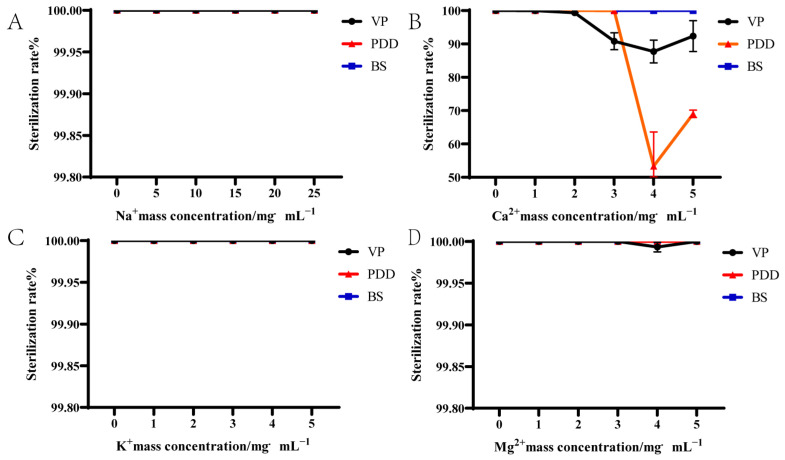
Effect of bactericidal effect of PHMB on different bacteria after addition of different ions. (**A**) Na^+^, (**B**) Ca^2+^, (**C**) K^+^, and (**D**) Mg^2+^.

We further treated the aquatic pathogens VP, PDD, and BS with the MBC of PHMB in the presence of additional Mg^2+^, Na^+^, and K^+^ ions (Figure 1). The latter provided no additional bactericidal benefits to PHMB; all three bacteria remained unchanged and bactericidal levels for all three bacteria exceeded 99% after 2 h of incubation. These data indicated that Na^+^, K^+^, and Mg^2+^ did not affect bactericidal activity. In contrast, the bactericidal activity of PHMB against VP and PDD were significantly reduced upon adding 2–5 mg/mL Ca^2+^ at the corresponding MBC. At a Ca^2+^ concentration of 4 mg/mL, the bactericidal levels against VP decreased to 87.73% and against PDD to 53.35%. However, Ca^2+^ did not affect the bactericidal activity of PHMB against BS (Figure 1). These results indicated no significant difference in the effect of Na^+^, K^+^, and Mg^2+^ on PHMB bactericidal activity against VP, PDD, and BS (*p* > 0.05). However, the bactericidal effect of PHMB on VP and PDD decreased with Ca^2+^ concentrations > 2 mg/mL and the response varied by strain.

### 3.3. Zeta Potential Changes Induced by PHMB

**Figure 2:**
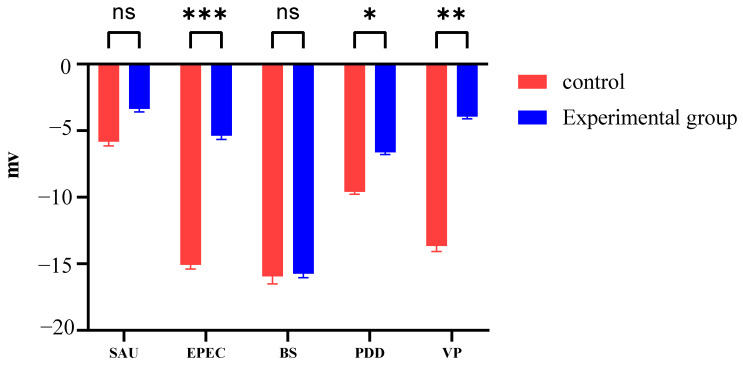
Changes in cell surface potential of SAU, EPEC, BS, PDD, and VP treated with PHMB (62.50 mg/mL). *** *p* < 0.001, ** *p* < 0.01, * *p* < 0.05, and ns, *p* > 0.05 no significant differences.

The zeta potential across bacterial species was also altered following treatment with 62.50 mg/mL PHMB and the Gram-positive strains exhibited a limited response (Figure 2): SAU levels were stable (−3.35 ± 0.20 mV vs. control −5.53 ± 0.25 mV, *p* > 0.05) while BS showed minimal change (−15.70 ± 0.31 mV vs. −15.90 ± 0.58 mV). In contrast, Gram-negative bacteria exhibited significant shifts in potential (*p* < 0.01) and the aquatic pathogens exhibited differential responses: VP displayed the most pronounced change (−3.92 ± 0.15 mV vs. control −13.70 ± 0.40 mV) followed by PDD (−6.81 ± 0.14 mV vs. −9.60 ± 0.18 mV, *p* = 0.09).

These different responses suggested that the interaction of PHMB at the membrane varies by species. Zeta potential modulation appears critical for the inactivation of Gram-negative bacteria, while Gram-positive species (SAU, BS) likely required additional mechanisms involving physiological and biochemical changes. 

### 3.4. PI Staining

**Figure 3:**
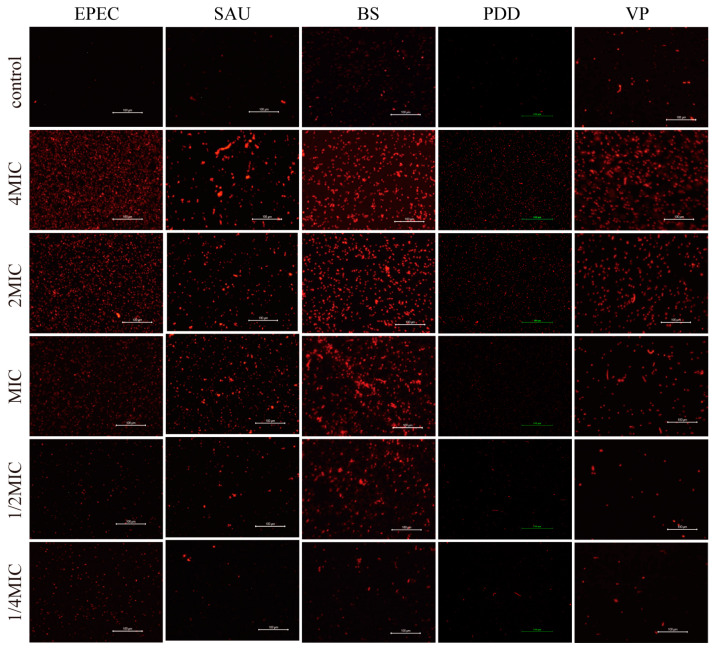
Fluorescent photomicrographs of PI staining of test bacteria treated at 4×, 2×, 1×, 1/2×, and 1/4 × MICs of PHMB. Magnification. 200×.

We also applied PI staining to bacterial cultures that had been treated with PHMB to assess viability (Figure 3). In general, all bacteria exhibited the strongest staining at 4 × MIC for PHMB, indicating a primary disruption of the bacterial cell membranes at this concentration. Staining signals were significantly reduced at 1/2× and 1/4 × MICs. As the concentration of PHMB decreased, staining signals also decreased, indicating that PHMB killed bacteria by disrupting their cell membranes. At higher PHMB concentrations, bacterial cell membranes were more disrupted compared to the low-concentration group. At 1/2× and 1/4 × MICs, bacterial staining signals were stronger than the control group but weaker than at 1×, 2× and 4 × MICs. This suggests that PHMB can still kill bacterial cells by disrupting cell membranes at lower concentrations, though the bacterial death rate is lower than at higher concentrations.

### 3.5. SEM Observation of Cell Morphology

**Figure 4:**
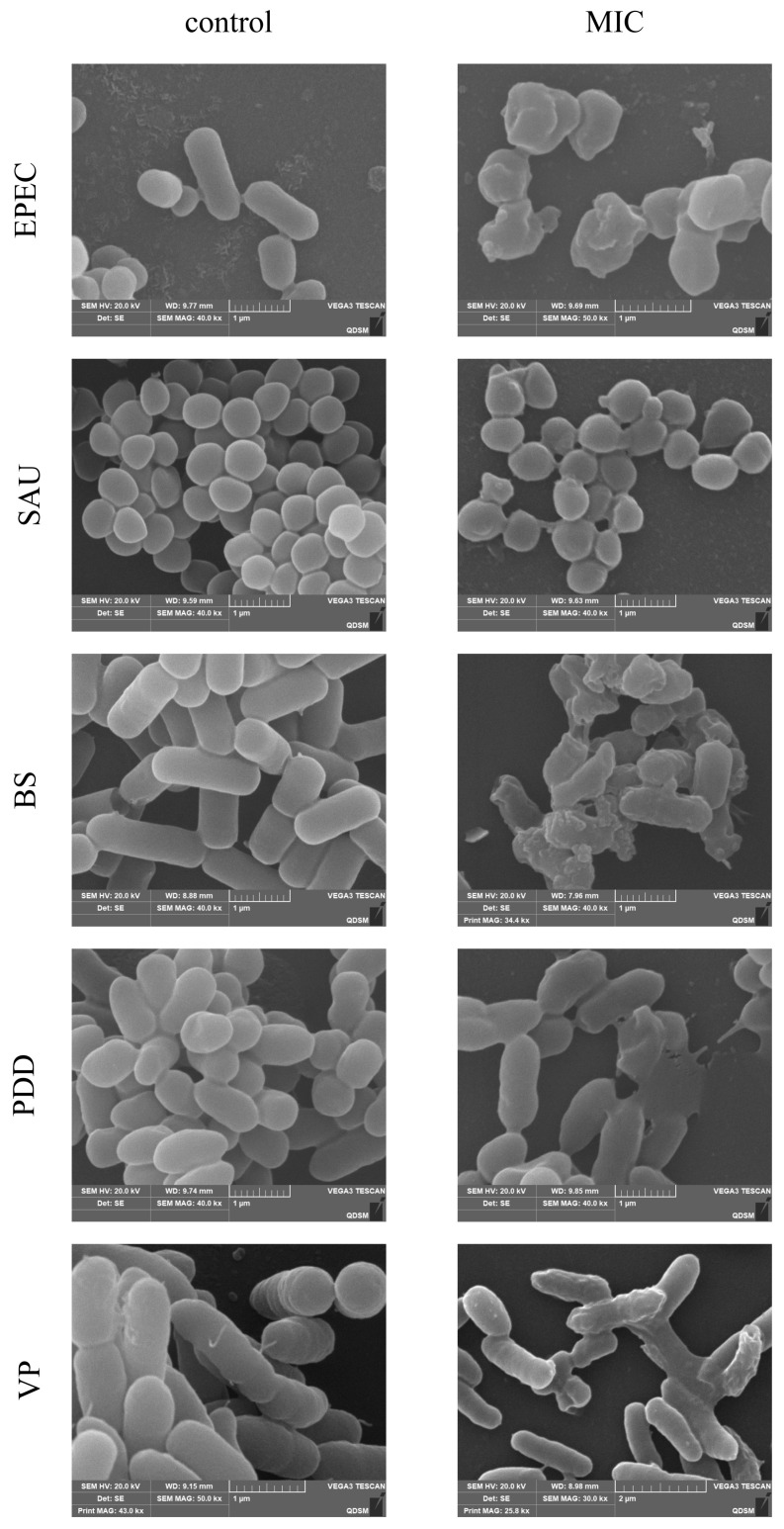
Electron photomicrographs of test bacteria following PHMB treatment at 1 × MIC for 1 h.

Bacterial cell morphology changes were confirmed by scanning electron microscopy that compared experimental and control groups across multiple bacterial strains (Figure 4). The EPEC control group possessed intact, smooth, and short cylindrical cells. In contrast, PHMB-treated cells were wrinkled, rough, and lacked the typical cylindrical shape with additional membrane structures. The SAU control group displayed smooth, wrinkle-free cells in spherical aggregates, whereas PHMB-treated cells exhibited air bubbles and dispersed cell structures. The BS control group had long, cylindrical cells with a smooth surface and minimal extracellular products. In contrast, PHMB-treated cells were wrinkled, with bubble formation and a shortened shape. The PDD control group had short, arc-shaped cells with a slightly uneven surface, while PHMB-treated cells were wrinkled and exhibited cell rupture and death. The VP control group possessed flat cells while PHMB-treated cells were rough and wrinkled.

Almost all bacteria exhibited cell wall rupture and crumpling at their respective 1 × MICs of PHMB. However, some cells remained well-preserved, with not all showing crumpling or rupture. These findings were consistent with the PI staining results. In contrast, SAU had the highest 1 × MICs among the strains, suggesting that higher PHMB concentrations are needed to induce cell rupture and death. The bacterial strains and their varying pathogenicity led to differences in PHMB-induced bacterial cell death at different concentrations.

### 3.6. DNA and RNA Leakage

**Figure 5:**
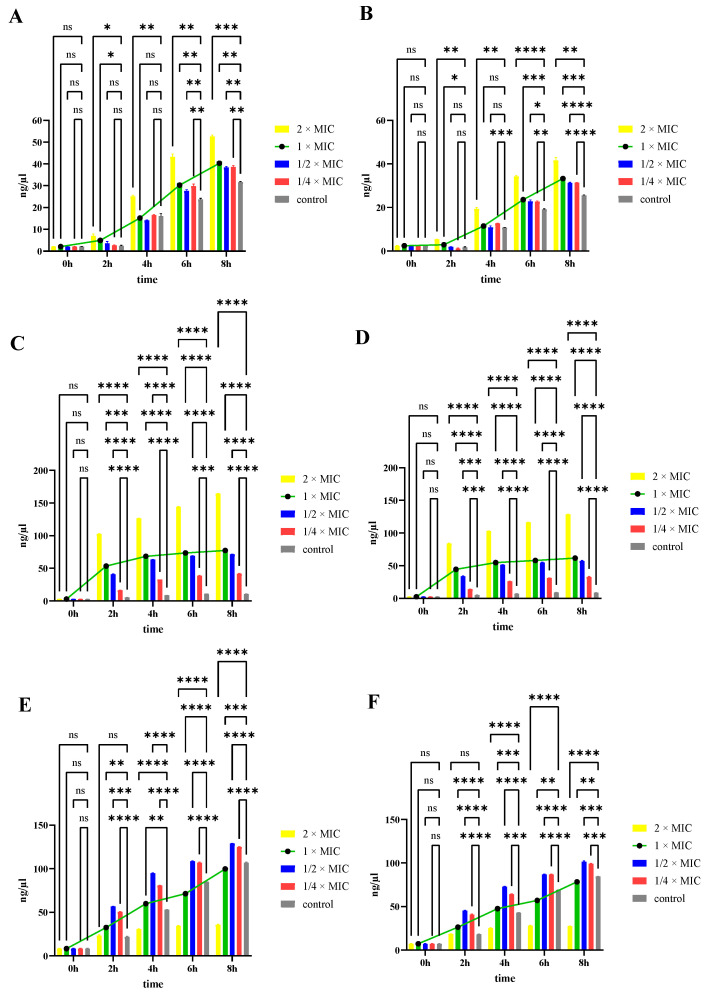
Effect of PHMB on cell permeability leading to intracellular DNA and RNA leakage. *V. parahaemolyticus* (**A**) DNA and (**B**) RNA leakage. *P. damselae* subsp. *damselae* (**C**) DNA and (**D**) RNA leakage. *B*. *subtilis* (**E**) DNA and (**F**) RNA leakage. ****p < 0.0001, *** *p* < 0.001, ** *p* < 0.01, * *p* < 0.05, ns, *p* > 0.05 no significant differences.

To observe the effects of PHMB treatment time and concentration on bacterial cell membrane permeability, extracellular DNA and RNA levels were measured after various treatment durations (Tables S1–S6 in Supplementary Material). Higher PHMB concentrations and longer treatment times led to increased extracellular DNA and RNA levels in VP and PDD. Significant differences in extracellular DNA and RNA were observed in VP treated with PHMB for 4 to 6 h compared to the control group, suggesting that prolonged PHMB treatment at the 1 × MIC induced substantial changes in cell membrane permeability (Figure 5A,B). PDD treated with 1 × MIC of PHMB for 2 to 4 h displayed significant differences in extracellular DNA and RNA compared to controls, indicating substantial changes in cell membrane permeability (Figure 5C,D). DNA and RNA leakage data indicated that PHMB was more effective at killing PDD than for VP. The extracellular DNA and RNA content in BS was significantly higher than in the control group at 1/4× and 1/2 × MICs. However, at 1× and 2 × MIC, DNA and RNA levels did not increase further. Combined with PI staining and scanning electron microscopy observations, increased PHMB concentrations resulted in more stained cells, altered cell permeability, and morphological changes such as wrinkling and deformation under electron microscopy, with cell rupture often observed. The reduced DNA and RNA leakage at 1 × MIC and 2 × MIC may be due to the failure to detect leakage at these concentrations.

### 3.7. The Prevention Experiment of PHMB on L. vannamei

**Figure 6:**
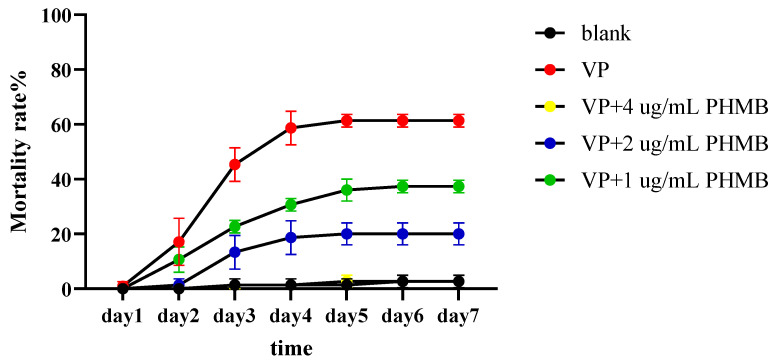
The mortality rate of *L. vannamei* during the process of treatment with different concentrations of PHMB after introducing 1 × 10^5^ CFU/mL of VP.

The mortality rates of *L. vannamei* in each group after adding VP and different concentrations of PHMB are shown in Figure 6. Only a few shrimps died in the 4 µg/mL treatment groups. The positive control group had the highest mortality rate, reaching 64% after stabilization. While the 1 ug/mL group also showed shrimp mortality, the mortality rate was lower than that of the positive group, reaching 37% after stabilization. The 2 ug/mL group only exhibited a small amount of mortality, reaching 20% after stabilization. These results suggest that PHMB provides the best protection against shrimp vibriosis.

## 4. Discussion

Cationic disinfectants are widely used and show promising research prospects due to their strong bactericidal activity and low biological toxicity [24]. Their effectiveness against aquatic bacteria, including *V. parahaemolyticus*, *V. campbellii,* and *V. owensii*, was found to vary [14]. In the current study, we found that PHMB exhibited varying levels of bactericidal activity on our test bacteria. PHMB demonstrated stronger bactericidal activity against Gram-negative bacteria (VP, PDD, and EPEC) but weaker activity against BS and SAU. The reasons for these effects were most likely due to the cell wall structure and membrane composition of Gram-positive bacteria [25]. Seawater is composed of about 96.5% pure water and 3.5% dissolved substances that primarily include Cl, Na, Mg, S, Ca, and K that account for >99% of the total dissolved substances [26]. The bactericidal effect of PHMB on pathogenic bacteria in seawater is influenced not only by the surface charge of the bacteria but also by its interaction with ions in seawater. We therefore examined whether other cations in the water alter the effectiveness of PHMB since the latter are positively charged. Na^+^, K^+^, and Mg^2+^ additions did not significantly alter the bactericidal effect of PHMB. In the available literature, Ca^2+^ reduced the bactericidal efficacy of PHMB against VP and PDD while having no effect on BS. Internal signal transduction relies on cyclic dinucleotides such as the 3′-5′ cyclic dimer GMP (c-di-GMP), where calcium acts as an environmental signal associated with c-di-GMP that influences biofilm formation [27]. Adding Ca^2+^ to the growth environment of VP promoted biofilm formation, increased cytotoxicity and lethal activity, and affected flagellar function to enhance bacterial motility. Additionally, calcium ions significantly up-regulated VP biofilm formation, virulence genes, and environmental adaptation genes [28]. This may explain why the addition of Ca^2+^ reduced the effectiveness of PHMB against PDD and VP.

The ionic composition of the bacterial cell surface is crucial for the function of PHMB. Due to the phospholipid composition of bacterial cells, the cytoplasmic membrane contains varying proportions of charged molecules, resulting in different surface charges across cells [29]. Drug effects on the bacterial cell surface potential correlates with its antimicrobial activity, and changes in surface potential can disrupt bacterial physiological functions and damage the membrane [30]. In general we found that PHMB treatment elevated the surface charge of the test bacteria, with similar effects using other cationic biocides having been previously reported [31]. Quaternary ammonium cationic surfactants (similar to PHMB) altered the zeta potential of bacterial surfaces with cations more likely to interfere with the outer membranes of Gram-negative bacteria [32]. Consistent with our findings, PHMB affected the surface potential of different bacteria differently, with minimal effect on Gram-positive bacteria. Along with the 1 × MIC results, the effect of PHMB on cell surface potential was not the sole pathway for structural disruption. Therefore, we further investigated the extent of PHMB-induced damage to the cell membrane.

PI staining is commonly used for bacterial viability staining since it only penetrates dead or membrane-damaged cells, where it stains DNA and RNA, making it a reliable indicator of membrane integrity [33]. We found a dose-dependent relationship with PHMB where higher concentrations caused greater membrane damage. The bactericidal effect of PHMB is not only cell membrane destruction but also the induction of physiological and biochemical changes in both the cell wall and membrane. The varying bactericidal effects are closely related to damage to both the cell wall and membrane.

SEM observations confirmed that PHMB induced distinct surface alterations in our test bacteria at 1 × MIC levels of PHMB. SAU cells presented raised surfaces while those of BS cells were wrinkled with prominent bulges; some cells also exhibited vacuole formation. EPEC cells appeared shortened and lysed, PDD cells showed rupture, and VP cells exhibited surface shrinkage and rupture. Our results were similar to previous findings using cationic surfactants that produced abnormal cell surface protrusions followed by lysis [34]. Blister formation on the bacterial surface has been shown to be the result of destabilization of the outer membrane by positive charges that facilitate membrane disruption by drugs. This process leads to intracellular membrane damage and loss of outer membrane integrity, resulting in blister formation [35]. 

Microorganisms can negatively impact aquacultural animals and lead to disease outbreaks [36]. The use of probiotics in aquatic environments is one of the most effective methods for preventing aquatic diseases and has been shown to be a scientifically sound means of controlling waterborne infections [37]. Seawater itself can inhibit pathogenic bacteria, but we found that the MICs of PHMB were similar between seawater pathogenic and probiotic bacteria. Therefore, we investigated DNA and RNA leakage from seawater microorganisms in the presence of PHMB to observe its effect on bacterial cell surfaces. Higher PHMB concentrations and longer exposure times correlate with increased DNA leakage. PI staining and SEM observations indicated that both the PHMB concentration and exposure duration were positively correlated with bacterial cell death. In BS cells, however, the concentration of DNA and RNA in the supernatant decreased after PHMB treatment. One reason for this is that PHMB can interact with extracellular nucleic acids to form insoluble complexes that precipitate at certain concentrations [38], with both nucleic acid length and PHMB concentration influencing precipitation [39]. DNA elongates and coils in solution, and as the coagulant concentration increases, the DNA strand undergoes a helix-to-sphere transition [40]. Polyvalent metal cations [41], polyamines [42], cationic and nonionic surfactants [43], as well as anionic liposomes and polymers [44] are DNA condensing agents. DNA was undetectable in BS after PHMB treatment, likely due to the length and structure of extracellular nucleic acids that may have been condensed with PHMB. However, PHMB had already disrupted the cell surface at the 1 × MIC.

PHMB has been applied in livestock, healthcare, and daily chemicals. It has also been proven to be safe for human use and for livestock products. For example, PHMB is safe for humans within the recommended concentration in existing daily chemical products [45]. For human skin wounds, PHMB has a safety profile similar to other commercially available cleaning and irrigation solutions. Furthermore, PHMB does not affect tissue viability or the production of pro-inflammatory cytokines [46]. This demonstrates that PHMB is non-toxic to humans when used for daily disinfection purposes. The previous literature has shown that the 48 h LC50 of PHMB for shrimp farming is 32.16 mg/L, which is much higher than the concentration of PHMB taken in the our experiment [47]. This paper investigated PHMB’s effectiveness in treating *L. vannamei* and its toxicity to aquatic animals. Animal experiments demonstrated that PHMB has a protective effect on aquaculture animals.

Common disinfectants used in aquaculture include chlorine-based disinfectants, halogen disinfectants, quaternary ammonium disinfectants, and hydrogen peroxide-based disinfectants. Chlorine-based disinfectants are inexpensive but are highly irritating and often result in the formation of disinfection by-products [48]. Halogen disinfectants hold an important position in the aquaculture industry due to their broad spectrum, high efficiency, rapid action, and relatively low environmental impact. However, they pose certain safety risks to aquatic animals [49]. Quaternary ammonium salts are widely used in aquaculture due to their good bactericidal effects, but some quaternary ammonium salts, such as benzalkonium bromide and cetyltrimethylammonium bromide, can promote the formation of bacterial biofilms [50,51]. PHMB is an effective bactericide with low cost and low toxicity to aquatic animals, making it a promising disinfectant for aquaculture. 

## 5. Conclusions

The results of this study demonstrate that PHMB effectively kills aquatic pathogens, such as VP and PDD, at low concentrations. A concentration of 15.63 µg/mL was sufficient to kill both VP at 1.0 × 10^6^ CFU/mL and PDD. PHMB is more effective against marine Gram-negative bacteria than Gram-positive bacteria. PHMB disrupts bacterial cell walls and membranes, resulting in changes in cell potential and surface morphology, thus inhibiting bacterial activity. Recent studies indicate that PHMB effectively controls harmful bacterial levels in pond water without affecting the survival of the probiotic BS. Therefore, in the field of bacterial disease control in aquaculture, PHMB, a novel cationic polymer biocide, holds significant potential for controlling shrimp AHPND and bacterial septicemia in fish (e.g., PDD infection).

## Figures and Tables

**Table 1:** MIC and MBC (µg/mL) of PHMB against bacterial test strains.

Bacteria †	MIC (µg/mL)	MBC (µg/mL)
EPEC	31.25	31.25
SAU	125.00	250.00
BS ‡	7.81	62.50
PDD ±	3.91	15.63
VP ±	7.81	15.63

† SAU, *S. aureus*; EPEC, *E. coli*; PDD, *P. damselae* subsp. *Damselae*; VP, *V. parahaemolyticus*; BS, aquatic *B. subtilis.* ± Aquatic pathogen. ‡ Probiotic bacterium.

## Data Availability

The data and references presented in this study are available from the corresponding author upon reasonable request.

## Supplementary Materials

The following supporting information can be downloaded at: https://www.mdpi.com/article/10.3390/biology14050470/s1, Table S1: The mean values of DNA leakage of different bacteria after VP was treated with different concentrations of PHMB (ng/µL), as well as the differences in the mean values. Table S2: The mean values of RNA leakage of different bacteria after VP was treated with different concentrations of PHMB (ng/µL), as well as the differences in the mean values. Table S3: The mean values of DNA leakage of different bacteria after PDD was treated with different concentrations of PHMB (ng/µL), as well as the differences in the mean values. Table S4: The mean values of RNA leakage of different bacteria after PDD was treated with different concentrations of PHMB (ng/µL), as well as the differences in the mean values. Table S5: The mean values of DNA leakage of different bacteria after BS was treated with different concentrations of PHMB (ng/µL), as well as the differences in the mean values. Table S6: The mean values of RNA leakage of different bacteria after BS was treated with different concentrations of PHMB (ng/µL), as well as the differences in the mean values.
