# Supplementary material for: Bactericidal Effect and Mechanism of Polyhexamethylene Biguanide (PHMB) on Pathogenic Bacteria in Marine Aquaculture
Source: Biology (Basel). 2025 Apr 25;14(5):470. doi: 10.3390/biology14050470 (PMC12109333; doi:10.3390/biology14050470)
Supplement: Supplementary file 1 [file biology-14-00470-s001.zip › biology-3560172-supplementary.pdf]

Table S1 The mean values of DNA leakage of different bacteria after VP was treated with different concentrations of PHMB (ng/µl), as well as the differences in the mean values.

	Average 1	Average 2	Mean Difference
	0h		
control vs. 2 MIC	2.080	2.080	0.000
control vs. MIC	2.080	2.080	0.000
control vs. 1/2 MIC	2.080	2.080	0.000
control vs. 1/4 MIC	2.080	2.080	0.000
	2h		
control vs. 2 MIC	2.403	6.993	-4.590
control vs. MIC	2.403	4.917	-2.513
control vs. 1/2 MIC	2.403	3.553	-1.150
control vs. 1/4 MIC	2.403	2.663	-0.2600
	4h		
control vs. 2 MIC	16.09	25.06	-8.970
control vs. MIC	16.09	15.20	0.8967
control vs. 1/2 MIC	16.09	14.08	2.017
control vs. 1/4 MIC	16.09	16.56	-0.4667
	6h		
control vs. 2 MIC	23.78	43.26	-19.47
control vs. MIC	23.78	30.26	-6.480
control vs. 1/2 MIC	23.78	27.63	-3.850
control vs. 1/4 MIC	23.78	29.76	-5.980
	8h		
control vs. 2 MIC	31.69	52.70	-21.01
control vs. MIC	31.69	40.32	-8.627
control vs. 1/2 MIC	31.69	38.37	-6.680
control vs. 1/4 MIC	31.69	38.70	-7.010

Table S2 The mean values of RNA leakage of different bacteria after VP was treated with different concentrations of PHMB (ng/µl), as well as the differences in the mean values.

	Average 1	Average 2	Mean Difference
	0h		
control vs. 2 MIC	2.440	2.440	0.000
control vs. MIC	2.440	2.440	0.000
control vs. 1/2 MIC	2.440	2.440	0.000
control vs. 1/4 MIC	2.440	2.440	0.000
	2h		

control vs. 2 MIC	1.653	5.313	-3.660
control vs. MIC	1.653	2.887	-1.233
control vs. 1/2 MIC	1.653	1.923	-0.2700
control vs. 1/4 MIC	1.653	1.143	0.5100
	4h		
control vs. 2 MIC	10.74	19.32	-8.587
control vs. MIC	10.74	11.49	-0.7500
control vs. 1/2 MIC	10.74	10.93	-0.1933
control vs. 1/4 MIC	10.74	12.70	-1.967
	6h		
control vs. 2 MIC	19.14	34.26	-15.12
control vs. MIC	19.14	23.57	-4.430
control vs. 1/2 MIC	19.14	22.76	-3.617
control vs. 1/4 MIC	19.14	22.66	-3.513
	8h		
control vs. 2 MIC	25.56	41.59	-16.04
control vs. MIC	25.56	33.25	-7.693
control vs. 1/2 MIC	25.56	31.35	-5.790
control vs. 1/4 MIC	25.56	31.36	-5.800

Table S3 The mean values of DNA leakage of different bacteria after PDD was treated with different concentrations of PHMB (ng/μl), as well as the differences in the mean values.

	Average 1	Average 2	Mean Difference
	0h		
control vs. 2 MIC	2.837	2.837	0.000
control vs. MIC	2.837	2.837	0.000
control vs. 1/2 MIC	2.837	2.837	0.000
control vs. 1/4 MIC	2.837	2.837	0.000
	2h		
control vs. 2 MIC	5.400	102.2	-96.84
control vs. MIC	5.400	53.46	-48.06
control vs. 1/2 MIC	5.400	40.90	-35.50
control vs. 1/4 MIC	5.400	16.53	-11.13
	4h		
control vs. 2 MIC	8.580	126.7	-118.1
control vs. MIC	8.580	68.48	-59.90
control vs. 1/2 MIC	8.580	63.30	-54.72
control vs. 1/4 MIC	8.580	32.81	-24.23
	6h		
control vs. 2 MIC	10.85	144.1	-133.2

control vs. MIC	10.85	73.33	-62.48
control vs. 1/2 MIC	10.85	69.14	-58.29
control vs. 1/4 MIC	10.85	38.56	-27.71
	8h		
control vs. 2 MIC	10.65	164.3	-153.7
control vs. MIC	10.65	77.33	-66.68
control vs. 1/2 MIC	10.65	71.35	-60.70
control vs. 1/4 MIC	10.65	41.71	-31.06

Table S4 The mean values of RNA leakage of different bacteria after PDD was treated with different concentrations of PHMB (ng/μl), as well as the differences in the mean values.

	Average 1	Average 2	Mean Difference
	0h		
control vs. 2 MIC	2.407	2.407	0.000
control vs. MIC	2.407	2.407	0.000
control vs. 1/2 MIC	2.407	2.407	0.000
control vs. 1/4 MIC	2.407	2.407	0.000
	2h		
control vs. 2 MIC	5.197	83.95	-78.76
control vs. MIC	5.197	44.36	-39.16
control vs. 1/2 MIC	5.197	33.66	-28.46
control vs. 1/4 MIC	5.197	13.69	-8.497
	4h		
control vs. 2 MIC	7.140	103.1	-95.93
control vs. MIC	7.140	54.76	-47.62
control vs. 1/2 MIC	7.140	51.23	-44.09
control vs. 1/4 MIC	7.140	26.16	-19.02
	6h		
control vs. 2 MIC	8.820	116.4	-107.5
control vs. MIC	8.820	58.00	-49.18
control vs. 1/2 MIC	8.820	54.95	-46.13
control vs. 1/4 MIC	8.820	31.04	-22.22
	8h		
control vs. 2 MIC	8.507	128.5	-120.0
control vs. MIC	8.507	61.61	-53.11
control vs. 1/2 MIC	8.507	57.27	-48.76
control vs. 1/4 MIC	8.507	32.79	-24.29

Table S5 The mean values of DNA leakage of different bacteria after BS was treated with different concentrations of PHMB (ng/μl), as well as the differences in the mean values.

	Average 1	Average 2	Mean Difference
	0h		
control vs. 2 MIC	8.293	8.293	0.000
control vs. MIC	8.293	8.293	0.000
control vs. 1/2 MIC	8.293	8.293	0.000
control vs. 1/4 MIC	8.293	8.293	0.000
	2h		
control vs. 2 MIC	21.71	23.37	-1.663
control vs. MIC	21.71	32.60	-10.89
control vs. 1/2 MIC	21.71	56.97	-35.27
control vs. 1/4 MIC	21.71	50.60	-28.89
	4h		
control vs. 2 MIC	53.20	30.72	22.48
control vs. MIC	53.20	59.99	-6.793
control vs. 1/2 MIC	53.20	94.98	-41.78
control vs. 1/4 MIC	53.20	80.77	-27.57
	6h		
control vs. 2 MIC	85.36	34.60	50.77
control vs. MIC	85.36	71.30	14.06
control vs. 1/2 MIC	85.36	108.7	-23.38
control vs. 1/4 MIC	85.36	107.0	-21.63
	8h		
control vs. 2 MIC	107.1	35.71	71.36
control vs. MIC	107.1	99.85	7.223
control vs. 1/2 MIC	107.1	129.0	-21.92
control vs. 1/4 MIC	107.1	125.0	-17.92

Table S6 The mean values of RNA leakage of different bacteria after BS was treated with different concentrations of PHMB (ng/μl), as well as the differences in the mean values.

	Average 1	Average 2	Mean Difference
	0h		
control vs. 2 MIC	7.297	7.297	0.000
control vs. MIC	7.297	7.297	0.000
control vs. 1/2 MIC	7.297	7.297	0.000
control vs. 1/4 MIC	7.297	7.297	0.000
	2h		
control vs. 2 MIC	18.45	18.64	-0.1833

control vs. MIC	18.45	26.38	-7.930
control vs. 1/2 MIC	18.45	45.49	-27.04
control vs. 1/4 MIC	18.45	41.00	-22.54
	4h		
control vs. 2 MIC	43.12	25.31	17.80
control vs. MIC	43.12	47.63	-4.513
control vs. 1/2 MIC	43.12	73.04	-29.92
control vs. 1/4 MIC	43.12	64.26	-21.14
	6h		
control vs. 2 MIC	69.29	28.29	41.00
control vs. MIC	69.29	57.04	12.25
control vs. 1/2 MIC	69.29	87.01	-17.71
control vs. 1/4 MIC	69.29	87.12	-17.82
	8h		
control vs. 2 MIC	84.63	27.68	56.94
control vs. MIC	84.63	78.44	6.187
control vs. 1/2 MIC	84.63	101.7	-17.09
control vs. 1/4 MIC	84.63	99.16	-14.53
